# The effects of acupuncture for patients with psoriasis: Study protocol for a randomized controlled trial

**DOI:** 10.1097/MD.0000000000026042

**Published:** 2021-05-28

**Authors:** Juan Du, Jiming Tao, Ming Xu, Runnan Wang, Lanmei Lin, Xinyun Huang, Qunyi Li, Xiaonian Lu

**Affiliations:** a Department of Dermatology, Huashan Hospital, Fudan University; b Department of Rehabilitation, Yueyang Hospital of Integrated Traditional Chinese and Western Medicine, Shanghai University of Traditional Chinese Medicine; c Shanghai Medical College, Fudan University; d Department of Acupuncture, Yueyang Hospital of Integrated Traditional Chinese and Western Medicine, Shanghai University of Traditional Chinese Medicine; e Department of Pharmacy, Huashan Hospital, Fudan University, Shanghai, China.

**Keywords:** acupuncture, anxiety, depression, psoriasis, quality of life

## Abstract

**Introduction:**

Psoriasis is a common chronic relapsing inflammatory skin disease, which may have considerable detrimental effects on the quality of life. Considering high costs and side effects associated with the use of conventional medications, acupuncture, as one of complementary and alternative nonpharmacological therapies, is commonly used in the management of psoriasis for reducing itching, repairing the skin lesions, etc. However, the effects of acupuncture in the management of psoriasis are still inconsistent, especially in psychosocial abnormality due to psoriasis. Therefore, we designed a randomized controlled trials (RCT) involving a placebo control to ensure participants’ blinding to investigate the effects of acupuncture for psoriasis in improving typical clinical symptoms and psychosocial abnormality.

**Methods:**

A singlecenter RCT was designed. 220 participants who meet the eligibility criteria will be randomly allocated into manual acupuncture group or sham acupuncture group in a 1:1 ratio. Participants will respectively receive 15 minutes manual acupuncture or sham acupuncture per session, 3 sessions per week, totally 12 weeks. Psoriasis Area and Severity Index scores, body surface area (BSA), Medical Outcomes Study 36-Item Short-Form Health Survey, Montgomery-Asberg Depression Rating Scale, and Depression, Anxiety, and Stress Scale-21 will be evaluated by blinded operators at baseline and 12 weeks. All analyses will be based on an intention-to-treat principle. The results will be published in an international peer-reviewed journal.

**Discussion:**

The results of this study are expected to clarify the effects of acupuncture on improving typical clinical symptoms and psychosocial abnormality of patients with psoriasis. It will contribute to clinical practice of acupuncture in the management of psoriasis.

**Trial registration:**

Chinese Clinical Trail Registry: ChiCTR2100045481. Registration date: April 17, 2021.

## 1 Introduction

Psoriasis is a common chronic relapsing inflammatory skin disease characterized by well-delineated red and scaly plaques, which is generally associated with genetic, immunological, metabolic, and endocrinal etiologies.^[1,2]^ With a prevalence rate of 2% to 4%, psoriasis, especially for plaque psoriasis, may have considerable detrimental effects on the quality of life.^[3]^ Patients with psoriasis also usually suffer from anxiety and depression due to pruritus symptoms, sleep disturbance, etc.^[4]^ Furthermore, patients with psoriasis may have a significantly increased risk of myocardial infarction and peripheral vascular disease because of accelerated atherosclerosis in an inflammatory state.^[5]^

Management of psoriasis still faces severe challenges. Several topical treatments, including topical corticosteroids, calcineurin inhibitors, immunosuppressants, biological agents, etc, are recommended to alleviate the clinical symptom to varying degrees.^[6]^ However, high costs and obvious side effects associated with the use of these medications limit their clinical application. They are not effective for anxiety, depression, and sleep disturbance of patients with psoriasis. Therefore, more and more patients with psoriasis are seeking therapeutic benefits of complementary and alternative therapies including traditional Chinese medicine, homeopathy, cognitive hypnotherapy, etc. especially on psychosocial abnormality.^[7]^

Acupuncture, as one of complementary and alternative nonpharmacological therapies, has been commonly used in the management of psoriasis, pruritus, and dermatitis in China.^[8]^ It is associated with a low risk of side effects and adverse reaction. The review reported that acupuncture may reduce itching by accelerating the microcirculation of the local lesion area, which is conducive to the extinction of inflammation and the absorption of metabolites.^[9]^ The study also reported acupuncture may improve the immune function of the body, stimulate the function of the meridian and repair the skin lesions, then prevent the progression to chronic recurrent seizures.^[10]^ Compared with conventional medications, the meta-analysis reported that acupuncture may show a potential advantage in the management of psychosocial abnormality including anxiety, depression, and so on.^[11,12]^ It also is associated with lower recurrence rate of psoriasis.^[13]^ However, the effects of acupuncture in the management of psoriasis are still inconsistent, especially in psychosocial abnormality due to psoriasis. Most of the results come from observational studies or case reports. There is poor methodological quality in published randomized controlled trials (RCT), especially in blinding. Therefore, we designed a RCT involving a placebo control to ensure participants’ blinding to investigate the effects of acupuncture for psoriasis in improving typical clinical symptoms and psychosocial abnormality.

## 2 Materials and methods

### 2.1 Trial design and ethics

A singlecenter RCT was designed to compare the effects of manual acupuncture and sham acupuncture in the management of psoriasis. The study has been approved by the ethics committee of the conducted hospital (No. KY2019–541). The study protocol follows the Standard Protocol Item Recommendations for Interventional Trails guidelines. This study protocol has been funded through the Chinese Clinical Trials Register. The registry number is ChiCTR2100045481, registration date: April 17, 2021.

**Figure 1:**
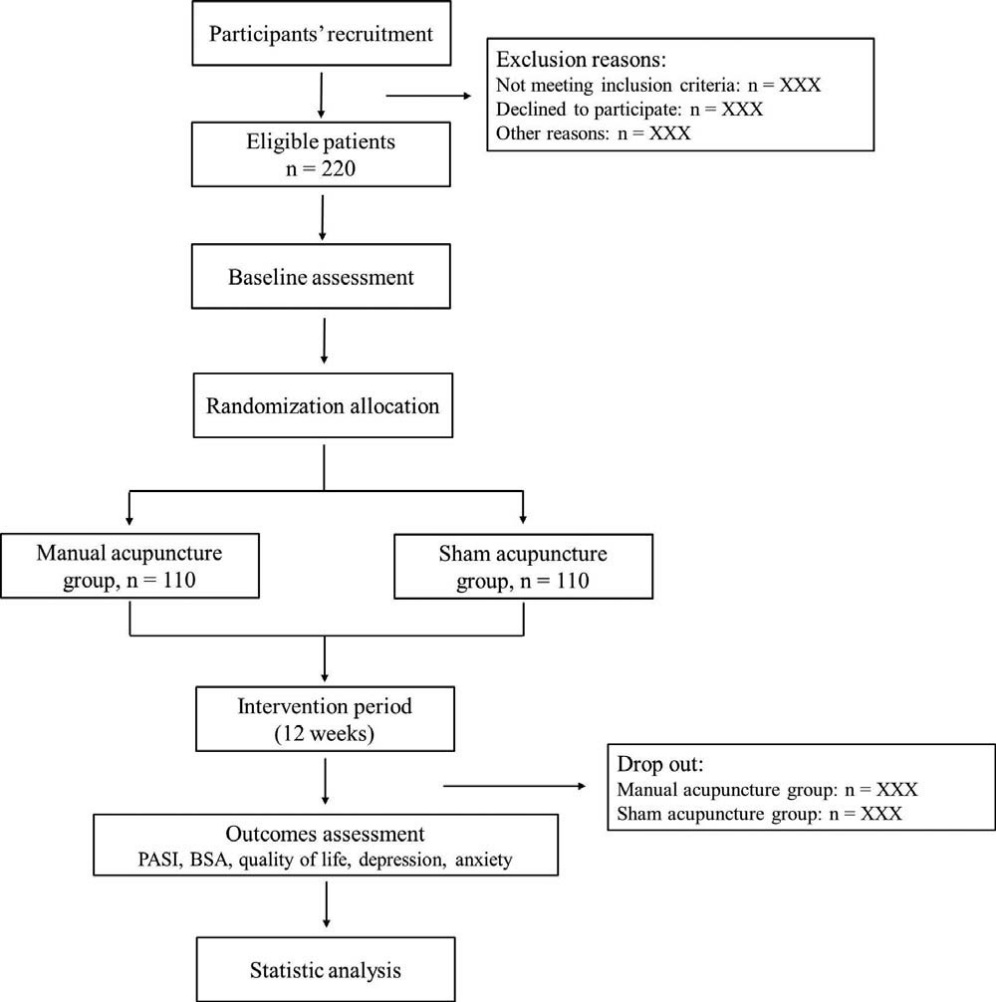
Flow diagram of participants.

In the study, 220 outpatients with psoriasis will be enrolled and be randomly allocated to manual acupuncture group (n = 110, expected) or sham acupuncture group (n = 110, expected). All participants will receive 36 sessions of treatments over 12 weeks. This study will be conducted in Huashan Hospital of Fudan University from October 2021 to December 2022. The study procedure is showed in Figure 1. The trial schedule is showed in Table 1.

**Table 1:** Trial processes chart.

Items	Before enrollment (week -1)	Intervention period (weeks 1–12)	End of intervention (week 13)
Inclusion criteria	√		
Exclusion criteria	√		
Informed consent	√		
Baseline measurement	√		
Randomization and allocation	√		
PASI	√		√
BSA	√		√
Quality of life	√		√
Depression	√		√
Anxiety	√		√
Adverse events			√

### 2.2 Patients

#### 2.2.1 Diagnostic criteria

The patients with psoriasis will be diagnosed according to the 2018 China College of Dermatology diagnostic criteria: skin lesions involve ≤15% body surface area (BSA). It should be mainly located on the torso/limbs, palm/soles, and face/scalp, the vulva area should not be unaffected.

#### 2.2.2 Inclusion criteria

The inclusion criteria for the study are as follows:

1. meeting the above diagnostic criteria for psoriasis;
2. aged between 18 and 65 years (either sex);
3. do not have history suggestive of contact allergy;
4. no acupuncture within 3 weeks prior to the recruitment;
5. refrained from using any other treatments from 3 weeks before acupuncture, without moisturizers;
6. agreeing to participate in the trial and be randomly allocated into any study groups.

#### 2.2.3 Exclusion criteria

Participants will be excluded who meet any following conditions:

1. other active skin diseases that may affect condition assessment;
2. severe psychological disorders;
3. pregnant or lactating women;
4. serious organic disease, systemic diseases, or other chronic diseases including cardiac disease, liver, or kidney disease, thrombocytopenia with bleeding tendency;
5. severe episodes of fainting during acupuncture or afraid of blood;
6. experiencing other active interventions that may affect the outcome of this study during the treatment;
7. any other condition that the investigators judge as likely to make the patient incapable to complete, comply, or unsuitable for the clinical trial.

#### 2.2.4 Recruitment

We intend to promote the recruitment plan through multimodal strategies, including advertisements on community bulletin boards, community broadcasts, social media (WeChat), and a clinical patient database. Researchers will register the interested participants and conduct a corresponding screening assessment. Informed consent will be obtained before randomization.

#### 2.2.5 Randomization and allocation

The participants will be randomly assigned to manual acupuncture group or sham acupuncture group at a 1:1 ratio after the baseline assessment. The randomization list will be generated using the Statistical Analysis Software (9.1) by a research assistant who is not involved in the recruitment of participants. The sealing and opaque envelopes will be used to achieve the allocation concealment.

#### 2.2.6 Blinding

The participants, statistical analysts and assessors will be blinded to treatment allocation. In order to blind the participants, the acupoints and manipulation of needles are similar in both groups. All participants will be asked whether they have experienced manual acupuncture or sham acupuncture after the treatments to assess the blinding. It will be impossible to blind the therapists due to the characteristics of acupuncture interventions.

### 2.3 Interventions

All participants will receive 36 sessions of 15-minutes duration over 12 weeks (3 sessions per week). Only trained acupuncturists will perform the acupuncture treatment, who must be instructed in standardized operating procedures including the locations of acupoints and the manipulation of needles,

#### 2.3.1 Manual acupuncture group

In the study, the acupuncture needles of 0.30 mm in diameter and 40 mm in length (Suzhou Hwato Medical Instruments Co. Ltd, China) will be used. All acupoints are identified based on the point location issued by the World Health Organization. The used acupoints include SP10 (Xuehai), LI11 (Quchi), ST36 (Zusanli), SP6 (Sanyinjiao), BL13 (Feishu), BL17 (Geshu), BL18 (Ganshu), BL20 (Pishu), and LR3 (Taichong). The insertion depth of SP10, LI11, ST36, and SP6 will be 1 cun. BL13, BL17, BL18, and BL20 will be punctured 1–15 cun into the skin, and LR3 will be punctured only 0.5 cun. The procedures for acupuncture are as follows:

1. skin disinfection;
2. the sterile adhesive pads will be placed on acupoints;
3. acupuncture manipulation is applied to inserted through the pads for Deqi sensation including soreness, numbness, distension, pain, and heaviness sensation;
4. the needles are maintained for 15 minutes for each session;
5. manual stimulation is conducted 3 times every 5 minutes during each session;
6. at end of each session, the needles are removed, and then clean cotton balls are pressed to avoid bleeding.

#### 2.3.2 Sham acupuncture group

Blunt-tipped placebo needles (0.30 mm in diameter and 40 mm in length, produced by Suzhou Hwato Medical Instruments Co. Ltd, China) will be used in the sham acupuncture group. The schedule, locations of acupoints, and other treatment settings are the same with manual acupuncture group but with superficial skin penetration and no needle manipulation for Deqi sensation. The needles will also be maintained for 15 minutes, and sham manual stimulation will be conducted each 5 minutes during each session.

### 2.4 Outcomes

#### 2.4.1 Primary outcomes

The primary outcome of the current study is the proportion of patients with a reduction in the Psoriasis Area and Severity Index (PASI) scores ≥50% compared with baseline. PASI score will be evaluated at the same or closet day. Target lesions will be recorded as digital photographs by single-lens reflex cameras during each session.

#### 2.4.2 Secondary outcomes

***BSA*** will be assessed by fingerprinting. The entire palm of the patient represents approximately 1% of the total BSA. The number of handprints on the psoriatic skin of the body part is used to determine the extent to which the body part is affected by psoriasis (%).^[14]^

***Quality of life*** will be assessed using the Medical Outcomes Study 36-Item Short-Form Health Survey, which consists of 36 items comprising 8 categories: functional capacity (10 items), physical aspects (4 items), pain (2 items), general health status (5 items), vitality (4 items), social aspects (2 items), mental health (5 items), and a question comparing current health conditions with those from a year ago. The total scores range from 0 to 100. High scores indicate more health status.^[15]^

***Psychosocial abnormalitydepression*** will be assessed by Montgomery-Asberg Depression Rating Scale, which consists of 9 items, each rated on a 7-step Likert scale from 0 to 6.^[16]^ Higher scores indicate greater severity. It is a useful measurement for depression in clinical practice, and has good internal consistency.^[17,18]^

Anxiety will be assessed using the Depression, Anxiety, and Stress Scale-21, which is recorded on a Likert-type scale ranging from 0 (not applicable at all) to 3 (applicable very much of the time). The severity of anxiety is graded into normal, mild, moderate, severe, or extremely severe based on the scores of each domain. The Depression, Anxiety, and Stress Scale-21 showed good validity and reliability.^[19]^

#### 2.4.3 Data collection and management

For the participants meeting the inclusion criteria, the research screeners will collect their baseline data at the appropriate time. The primary and secondary outcomes will be assessed by the blinding assessors at baseline and 13 weeks (within 1 week after the intervention). The research assistant, who is blind to the allocation, will complete the quality control of data collection and data entry. The research manager will complete the collection, classification, identification, and analysis of raw data.

### 2.5 Statistical analysis

#### 2.5.1 Clinical data analysis

In this study, clinical data will be analyzed using SPSS 19.0 statistical software by a blinded statistician. Data analysis will also be based on the intention-to-treat. Continuous variables will be showed using the mean and standard deviation, or the median and interquartile range. Categorical variables will be presented with percentages (%). A two-sample *t* test will be used in comparing clinical data between manual acupuncture group and sham acupuncture group. A paired sample *t* test will be used for comparisons between baseline and the end of treatment in each group. Clinical data on the skewed distribution will be compared using a nonparametric test. Chi-Squared test or Fisher exact test will be used for comparing categorical variables. The significance level of the test is targeted at 0.05.

#### 2.5.2 Sample size

Based on the PASI scores, the published study reported that the total curative rate in the acupuncture group was 97.5%, and 87% in the control group.^[20]^ In the current study, we assumed that a significance level α was 0.05 and a power (1-β) = 0.80, and calculated the sample size: 88 participants with psoriasis would be required in each group for 80% power to detect a difference between manual acupuncture group and sham acupuncture group using the two-sided Z test with pooled variance. The significance level of the test is 0.05. Given a 20% loss to follow-up, therefore this study will require at least 220 participants.

### 2.6 Quality control and safety monitoring

In order to complete the trial with high quality, the recruitment must be strictly in accordance with the inclusion/exclusion criteria of the trial protocol. The participants must be randomly grouped using allocation sequence generated by the software statistical analysis software 9.1. The participants are free to withdraw from this trial at any time for any reason to ensure data authenticity and credibility. Of cause, the researchers will try their best to get the data of last evaluation for an intention-to-treat analysis.

The acupuncturists are graduated from acupuncture and moxibustion major in the University of Traditional Chinese Medicine, who are trained in aseptic acupuncture procedures again. The researchers will document, report and analysis any adverse event including subcutaneous hematoma, fainting, and so on during every session. Before and after the study, all participants will receive liver and kidney function examinations, routine blood texts, and stool tests to exclude severe heart/liver/kidney diseases and evaluate possible side effects of the acupuncture.

## 3 Discussion

As a chronic, inflammatory, immune-mediated skin disease, psoriasis is characterized by frequent relapses.^[21]^ It usually requires combination medicines for alleviating rash in the clinic, but this does not always result in satisfactory long-term effects. The studies shown that the 48-week recurrence rate in patients treated with ixekizumab was as high as 87%,^[22]^ 3-month recurrence rate in patients treated with brodalumad (an interleukin-17 targeted biological) was 78%,^[23]^ 4-month recurrence rate in patients treated with narrow-spectrum ultraviolet B was 54.55%.^[24]^ Therefore, there is increasing concern regarding the complementary and alternative medicine in the modern practice of various dermatology.

According to the theory of traditional Chinese medicine, psoriasis should be mostly a “blood stasis” syndrome. Acupuncture may promote blood circulation, remove blood stasis, and regulate qi and blood circulation. The studies showed that acupuncture have beneficial effects for the treatment of plaque psoriasis.^[13,25]^ Although the mechanism of acupuncture for psoriasis is not clear, there are indications that acupuncture combined with other treatments, including western medicine, phototherapy, and so on, can alleviate plaque psoriatic skin inflammation and excessive thickening of skin lesions.^[26]^ However, the effect of acupuncture alone for psoriasis is less evaluated especially comparing with sham acupuncture. In addition, psoriasis may also be associated with psychological disorders.^[27]^ It seriously affects the quality of life and is an economic burden to the patient. Previous studies reported that acupuncture showed a potential advantage in the management of anxiety and depression.^[11,12]^ Therefore, the current study will evaluate the effect of manual acupuncture in the management of psoriasis without the compounding interference of combination treatment strategies.

### 3.1 Limitation

There still are limitations in the current study. First, it will be impossible to blind the therapists due to the characteristics of acupuncture interventions. In order to minimize the risk of detection bias, all results will be assessed by independent researchers. Second, small deviations in the manipulation of acupuncture therapy and locations of acupoints are inevitable, but performance bias can be reduced through unified professional training for acupuncturists. Third, patients choosing alternative therapies may be inclined to choose manual acupuncture treatment, which result in allocation bias during recruiting patients for the sham acupuncture group. In conclusion, this study aims to verify whether manual acupuncture therapy is effective for psoriasis. We also hope to provide a safe and effective treatment for psoriasis through this study.

## Author contributions

**Conceptualization:** Juan Du, Jiming Tao, Qunyi Li, Xiaonian Lu.

**Funding acquisition:** Qunyi Li, Xiaonian Lu.

**Methodology:** Juan Du, Ming Xu, Runnan Wang, Qunyi Li.

**Project administration:** Juan Du, Lanmei Lin, Xinyun Wang, Xiaonian Lu.

**Writing – original draft:** Juan Du, Jiming Tao, Lanmei Lin, Xinyun Wang.

**Writing – review & editing:** Runnan Wang, Xiaonian Lu.
